# Transposon Mutagenesis Identifies Novel Genes Associated with *Staphylococcus aureus* Persister Formation

**DOI:** 10.3389/fmicb.2015.01437

**Published:** 2015-12-23

**Authors:** Wenjie Wang, Jiazhen Chen, Gang Chen, Xin Du, Peng Cui, Jing Wu, Jing Zhao, Nan Wu, Wenhong Zhang, Min Li, Ying Zhang

**Affiliations:** ^1^Key Laboratory of Medical Molecular Virology, Huashan Hospital, Shanghai Medical College of Fudan UniversityShanghai, China; ^2^Department of Laboratory Medicine, Huashan Hospital, Shanghai Medical College of Fudan UniversityShanghai, China; ^3^Department of Laboratory Medicine, School of Medicine, Renji Hospital, Shanghai Jiao Tong University School of MedicineShanghai, China; ^4^Department of Molecular Microbiology and Immunology, Johns Hopkins Bloomberg School of Public Health, Johns Hopkins UniversityBaltimore, MD, USA

**Keywords:** persisters, *Staphylococcus aureus*, antibiotics, transposon mutant library, stress conditions

## Abstract

Pathogenic bacterial persisters are responsible for the recalcitrance of chronic and persistent infections to antimicrobial therapy. Although the mechanisms of persister formation and survival have been widely studied in *Escherichia coli*, persistence mechanisms in *Staphylococcus aureus* remain largely unknown. Here, we screened a transposon mutant library of a clinical methicillin-resistant *Staphylococcus aureus*(MRSA)strain, USA500 (ST8), under antibiotic pressure and identified 13 genes whose insertion mutations resulted in a defect in persistence. These candidate genes were further confirmed by evaluating the survival of the mutants upon exposure to levofloxacin and several other stress conditions. We found 13 insertion mutants with significantly lower persister numbers under several stress conditions, including *sdhA, sdhB, ureG, mnhG1, fbaA, ctaB, clpX, parE, HOU_0223, HOU_0587, HOU_2091, HOU_2315, and HOU_2346*, which mapped into pathways of oxidative phosphorylation, TCA cycle, glycolysis, cell cycle, and ABC transporters, suggesting that these genes and pathways may play an important role in persister formation and survival. The newly constructed knockout strains of *ureG, sdhA* and *sdhB* and their complemented strains were also tested for defect in persisters following exposure to levofloxacin and several other stress conditions. The results from these experiments were consistent with the screening results, which indicated that deletion of these genes in MRSA USA500 leads to persister defect. These findings provide novel insights into the mechanisms of persister formation and survival in *S. aureus* and offer new targets for the development of persister-directed antibiotics for the improved treatment of chronic and persistent infections.

## Introduction

Bacterial persisters were first described by Hobby in 1942 (Hobby et al., 1942) and have since been identified in various bacterial pathogens, such as *Escherichia coli, Staphylococcus aureus, Mycobacterium tuberculosis*, and *Pseudomonas aeruginosa* (Moyed and Bertrand, 1983; Mulcahy et al., 2010; Zhang et al., 2012). Persisters are a small bacterial population that survive a lethal concentration of antibiotics (Lewis, 2010). This heterogeneous subpopulation of slow- or non-growing bacterial cells regrow after removal of the antibiotic (Zhang, 2014). Studies have confirmed that persisters are the main reason for the recalcitrance of chronic and persistent infections to antimicrobial therapy, especially infections involving *S. aureus* and *P. aeruginosa* (Kint et al., 2012). *S. aureus* is an opportunistic pathogen that can cause severe wound and nosocomial infections. In particular, methicillin-resistant *S. aureus* (MRSA) is a major threat in hospital and community settings. Consequently, persisters pose significant challenges for the effective treatment of various infections.

The mechanisms of persister formation and survival have been studied mainly in Gram-negative bacteria, such as *E. coli*, and various genes and pathways have been confirmed to be involved in persister formation or survival (Zhang, 2014). The best-known pathways include toxin-antitoxin modules (HipA/B) (Moyed and Bertrand, 1983), SOS response/ DNA repair (LexA) (Debbia et al., 2001), energy production (SucB, UbiF) (Ma et al., 2010), stringent response (RelA) (Korch et al., 2003), phosphate metabolism (PhoU) (Li and Zhang, 2007), and the trans-translation mediated pathway (SsrA and SmpB) (Li et al., 2013). However, the mechanisms of persistence in Gram-positive pathogens such as *S. aureus* remain largely unknown. Stationary cultures of *S. aureus* ATCC55585 were shown to be tolerant to ciprofloxacin (Keren et al., 2004). Several genes have been confirmed to be associated with persisters in *S. aureus*, including *hemB, mazF* and *glpK* (Fu et al., 2009; Singh et al., 2009; Han et al., 2014). In addition, *S. aureus* biofilms display high tolerance to antibiotics due to the existence of persisters (Singh et al., 2009). Drug tolerance is determined by pre-existing persisters and adaptive responses after drug exposure (Lechner et al., 2012b). Due to the significant differences between Gram-positive and Gram-negative bacterial cells in terms of cell structure, gene modulation and stress response, the mechanisms of persister activity could differ between these organisms and thus warrant detailed studies.

To understand the mechanisms of persister formation and survival in *S. aureus*, we screened a transposon mutant library of the CA-MRSA clinical isolate USA500 for responses to antibiotic pressure and identified 13 genes whose mutations cause defect in persister formation.

## Materials and methods

### Bacterial strains, plasmids, antibiotics, and growth conditions

*E. coli* strains DH5α and TOP10 and *S. aureus* strain RN4220 were used in DNA cloning. The *S. aureus* USA500, MRSA ST8 clinical wild-type strain was used as a parent strain for screening. The plasmids pRB473 and pKOR1 were used for genetic complementation and homologous recombination, respectively (Bae and Schneewind, 2006). Antibiotics were used at the following concentrations, unless otherwise indicated: erythromycin, 5 μg/ml; ampicillin, 100 μg/ml; chloramphenicol, 10 μg/ml; levofloxacin, 12.5 μg/ml; and anhydrotetracycline (ATc), 1 μg/ml. Luria-Bertani (LB) broth or agar was used as the culture medium for *E. coli*, and tryptic soy broth (TSB) or tryptic soy agar (TSA) was used for *S. aureus* growth.

### MIC and MBC determination

The MIC (minimum inhibitory concentrations) and MBC (minimum bactericidal concentrations) of different antibiotics against the parent strain *S. aureus* USA500 and its mutants were determined as described previously (Murray, 1999). All MIC and MBC assays were repeated at least three times.

### Drug-exposure screen of transposon mutant library screen and inverse PCR identification of mutated genes

The *S. aureus* strain USA500 was subjected to transposon mutagenesis using a previously described method (Li et al., 2009). The mariner-based transposon mutagenesis exhibited no bias which was validated by inverse PCR. The transposon mutant library, which consisted of 9120 clones, was grown in TSB medium containing 5 μg/ml erythromycin in 96-well plates overnight at 37°C. The library was inoculated (1:100) into fresh TSB medium using a 96-well replicator (Sigma, USA) and incubated in the 96-well plates at 37°C for 16 h to allow the bacteria to grow to the stationary phase. Levofloxacin was added to each well at a final concentration of 12.5 μg/ml. The drug-exposed cultures were replica-transferred onto TSA plates after 3 days and 6 days of drug exposure at 37°C.

Mutants that displayed higher susceptibility to levofloxacin relative to the parent strain after 3 or 6 days of drug exposure were selected, and the drug exposure assay was repeated to confirm the stability of the phenotype. Mutants with a decreased survival phenotype in both screening rounds were selected for identification.

Two rounds of inverse PCR were performed to locate the transposon insertions in the *S. aureus* mutants. The overnight culture of each mutant was lysed in lysis buffer (Takara, Japan) followed by boiling at 100°C for 10 min. The supernatant was used as the template for the inverse PCR. The PCR primers are listed in Table S2. The first round of PCR was performed in a final volume of 25 μl. The first-round PCR cycling parameters were as follows: 95°C for 5 min; 6 cycles of 94°C for 30 s, 30°C for 30 s, and 72°C for 1 min; 30 cycles of 94°C for 30 s, 45°C for 30 s, and 72°C for 1 min; and 72°C for 5 min. PCR (2 μl) products were used as the template for the second round of PCR, which was performed at 95°C for 5 min, followed by 35 cycles of 45°C for 30 s and 72°C for 1 min, and finally 72°C for 5 min. The PCR products of the second round were sequenced using the respective “erm” internal primers (Table S2). The insertion alleles were identified by a BLASTn search. Since USA500 was the progenitor of USA300, the USA300 genome was used as a reference.

### Allelic gene replacement and genetic complementation

To knockout the candidate persistence genes *sdhA, sdhB*, and *ureG* from the genome of USA500 wild-type, the plasmid pKOR1 was used to perform homologous recombination (Table S2). The allelic recombination procedure was performed as described previously (Bae and Schneewind, 2006). DNA regions located 1 kb upstream and downstream of the candidate genes were PCR-amplified from the chromosomal DNA of the parent strain USA500. The PCR products of the candidate genes were ligated and cloned into pKOR1 by recombination reactions (BP clonase enzyme mix, Invitrogen). The resulting plasmids were transferred to RN4220 cells and subsequently to the parent strain USA500 via electroporation. The allele replacement was performed using the following two steps: the plasmid was integrated into the chromosome by incubating the cultures at 43°C in TSB with 10 μg/ml chloramphenicol (TSB_cm10_), and plasmid eviction was performed at 30°C in TSB_cm10_. The cultures were diluted 10^4^ times with sterile water. Then, 100 μl of the diluted culture was spread on TSA containing 0, 1, or 2 μg/ml anhydrotetracycline (ATc) and incubated at 30°C for 2 days. The candidate deletion mutants were picked from TSA_ATc1_ and incubated in TSB_cm10_ at 37°C overnight. Finally, chromosomal DNA was purified from the candidate mutants, which were then confirmed by PCR amplification and DNA sequencing. The primers gene-att1, gene-att2, gene-rev1, and gene-rev2 were used for allelic recombination. After the candidate genes were knocked-out, complementation strains were constructed using the shuttle vector pRB473. The candidate genes with 200 bp upstream from the ORF which contained the predicted promoters were PCR-amplified and then cloned into pRB473. The promoters were amplified by PCR with primers Pro-F and Pro-linker, and the genes were amplified with primers gene-linker and gene-R (Table S2). The recombinant plasmids were transformed into the gene deletion mutants via electroporation.

### Persister assays

To verify the defect in persistence to levofloxacin, the knockout mutants, complemented strains, and the parental strain were cultured to stationary phase (18–20 h) in TSB without shaking. A final concentration of 12.5 μg/ml levofloxacin was added to 1 ml of the stationary-phase bacteria in 1.5-ml tubes, which were then incubated at 37°C without shaking. The TSA plates were used for CFU counting on days 1 to 6 by 10-fold serial dilutions.

### Susceptibility to other stresses in exposure assays

For the heat stress assay, stationary-phase cultures of the knockout mutants, complemented strains, and the parent strain were incubated at 57°C in a water bath for up to 3 h; the number of CFU was then determined by plating serial dilutions of the bacteria on TSA plates.

For the oxidative stress test, stationary-phase cultures were diluted 1:100 with TSB and exposed to hydrogen peroxide (H_2_O_2_) at a final concentration of 50 mM for up to 4 h. The survival of bacteria was determined at different times by plating serial dilutions on TSA plates.

For the acid stress test, the TSB was adjusted by adding HCl until the medium reached a pH of 3.0 and was filter-sterilized. The stationary phase cultures were harvested and washed with TSB at pH 3.0. Then, the cells were resuspended in the pH-adjusted TSB medium and incubated at 37°C without shaking. The survival of the bacteria was determined after 0, 1, 2, and 3 days. Control samples were subjected to the same treatment except that TSB at pH 7.0 was used. For the organic acid (acetic acid, pH 4.4) stress test, stationary-phase cultures were diluted 1:100 with TSB containing acetic acid at a final concentration of 80 mM. The cultures were incubated at 37°C without shaking for 1, 2, and 3 days. The survival of bacteria was determined by plating serial dilutions on TSA plates.

### Statistical analysis

All persister assays were repeated at least three times in this study. The data points in the graphs represent the average of three independent experiments, and error bars represent the standard deviations. A Student's *t*-test was performed for pairwise comparison, and *P*-value < 0.05 was considered statistically significant.

## Results

### Screening for transposon mutants with reduced persister levels

**Figure 1:**
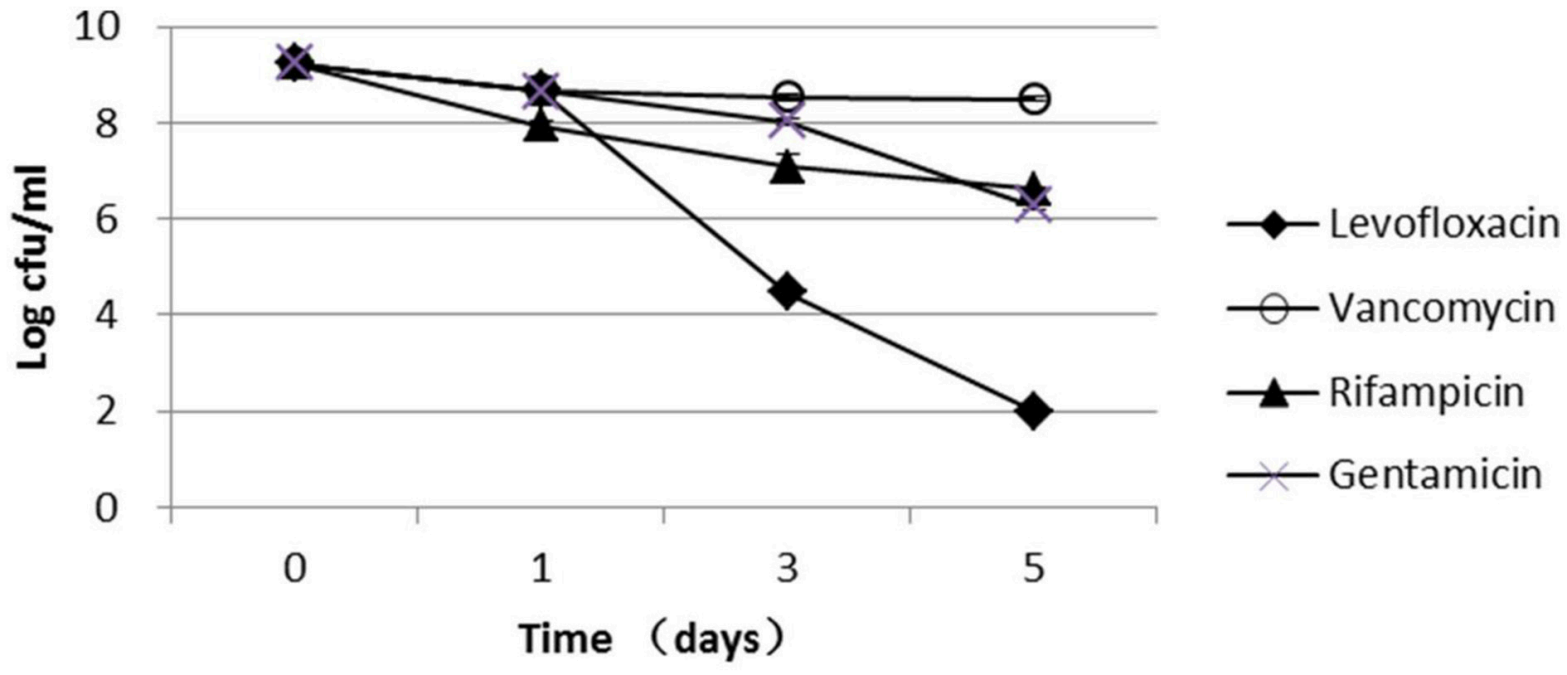
**Persister levels of the parent *S. aureus* strain USA500 in drug exposure assays**. Stationary-phase cultures of the wild-type USA500 were exposed to the different drugs, including levofloxacin (12.5 μg/ml), vancomycin (25 μg/ml), rifampicin (1.5 μg/ml), and gentamicin (100 μg/ml). The vertical axis represents CFU values in log scale, and the horizontal axis represents the time of exposure. The error bars indicate standard deviations.

The parent *S. aureus* strain used in this study, USA500, is a clinical isolate that is resistant to penicillin, oxacillin, cefazolin, cefuroxime, and cefoxitin and is susceptible to gentamicin, vancomycin, linezolid, levofloxacin, and rifampicin (Table S1). Because vancomycin, rifampicin, and gentamicin had very low bactericidal activity against the stationary-phase cultures, these antibiotics were not used for persister screening in this study (Figure 1). Levofloxacin had a high bactericidal activity against stationary-phase bacteria, which were decreased by 5-log and 7-log after 3- and 5-day exposure, respectively (Figure 1); therefore, levofloxacin was used to screen for mutants with reduced persister numbers. Because levofloxacin concentrations from 12.5 μg/ml (50 × MIC) to 50 μg/ml (200 × MIC) had almost the same killing rate (Figure S1), 12.5 μg/ml levofloxacin was selected for the mutant screening in this study.

Among the 9120 clones in the transposon mutant library, 260 were identified as having a decreased survival phenotype after 6 days of levofloxacin exposure in two rounds of screening (Figure S2). The clones exhibiting decreased survival phenotypes in the first round of screening were chosen for inclusion in the second round of screening under the same condition to ensure the accuracy of screening results. All 260 mutant clones were subjected to inverse PCR and DNA sequencing, which resulted in the identification of 13 candidate genes: *sdhA, sdhB, ureG, mnhG1, ctaB, fbaA, clpX, parE, HOU_0223, HOU_0587, HOU_2091, HOU_2315*, and *HOU_2346* (Table 1). Importantly, *sdhA, sdhB, mnhG1*, and *HOU_0587* were identified from multiple clones in more than one mutant with different insertion sites, which strongly indicates their involvement in persistence. These genes belonged to pathways of oxidative phosphorylation, TCA cycle, glycolysis, cell cycle, and ABC transporters by KEGG pathway annotation.

**Table 1:** **Candidate genes whose mutations are associated with defect in persister formation identified from the transposon mutant library of *S. aureus* USA500**.

**Genes**	**Gene products**	**Number of insertions**
*HOU_2091*	Hypothetical membrane protein	>5
*HOU_2315*	Lysostaphin resistance protein A	>5
*sdhA*	Succinate dehydrogenase flavoprotein subunit	3
*sdhB*	Succinate dehydrogenase iron-sulfur subunit	2
*mnhG1*	Monovalent cation H+ antiporter subunit G	2
*HOU_0587*	Putative pyridine nucleotide-disulfide oxidoreductase	2
*ureG*	Urease accessory protein UreG	1
*ctaB*	Protoheme IX farnesyltransferase	1
*HOU_0223*	Maltose ABC transporter permease	1
*fbaA*	Fructose-bisphosphate aldolase	1
*HOU_2346*	Response regulator of the LytR AlgR family	1
*clpX*	ATP-dependent protease ATP-binding subunit ClpX	1
*parE*	DNA topoisomerase IV, B subunit	1

All mutant clones with decreased survival phenotype were subjected to inverse PCR and DNA sequencing, and the 13 candidate genes were identified and are listed in this Table. Insertion sites indicate the number of identified insertion sites in a given gene from different mutants.

Mutations with increased antibiotic susceptibility could also have decreased survival under antibiotic pressure. To exclude this possibility, we examined the MIC and MBC of levofloxacin for these mutants. The results showed that the MIC and MBC values of all the insertion mutants were unchanged compared with the parent strain (data not shown), suggesting that these genes are not involved in antibiotic resistance.

### The candidate mutants are more susceptible to a variety of stresses

Because genes involved in antibiotic persistence could also contribute to stress persistence (Li and Zhang, 2007; Ma et al., 2010), we tested the survival of the candidate mutants upon exposure to heat and acidic pH compared with the parent strain. As measured in the heat (57°C) and acid stress (pH 3.0) assays, *ureG, sdhA, ctaB, sdhB, clpX, mnhG1*, and *HOU_0587 mutants* were found to be more susceptible to the stresses than the parent strain (Table 2). Under acid stress, the *ureG, sdhA, ctaB, sdhB*, and *HOU_0587* mutants were among the most susceptible mutants and showed a nearly 4-log decrease in CFU compared with the parent strain after 3 days of treatment. The *clpX, mnhG1* and *HOU_0223* mutants showed 1-2 log decreases after 3 days of treatment. Similarly, under heat stress, the *ureG, sdhB, ctaB, clpX*, and *mnhG1* mutants exhibited a 1–2 log decrease in persister levels after 2 h of heat stress compared with the parent strain, and *sdhA, parE*, and *HOU_2091*displayed nearly 1-log decrease in persister levels. The *fbaA, HOU_0223, HOU_2315*, and *HOU_234*6 mutants were not affected by either heat or acid treatment. None of the unstressed controls for the mutants or the parent strain showed decreased survival after 3 days.

**Table 2:** **Persister deficiency of the identified mutants in exposure to different stress conditions**.

**Genes**	**No. of bacteria (Log CFU/ml, mean ± SD)**							
	**pH 3.0**				**57°C**			
	**Start**	**1 d**	**2 d**	**3 d**	**0.5 h**	**1.0 h**	**1.5 h**	**2.0 h**
USA500	9.7 ± 0.15	7.8 ± 0.17	7.0 ± 0.22	4.8 ± 0.16	7.4 ± 0.23	5.7 ± 0.24	4.7 ± 0.19	4.1 ± 0.17
*ureG*	9.4 ± 0.15	6.2 ± 0.28^*^	2.8 ± 0.17^*^	1.0 ± 0.15^*^	6.0 ± 0.22^*^	3.6 ± 0.20^*^	2.6 ± 0.27^*^	2.0 ± 0.17^*^
*sdhA*	9.6 ± 0.18	6.8 ± 0.22^*^	5.5 ± 0.18^*^	1.4 ± 0.22^*^	6.9 ± 0.27	5.7 ± 0.13	4.6 ± 0.15	3.5 ± 0.13^*^
*sdhB*	9.3 ± 0.16	6.3 ± 0.23^*^	3.7 ± 0.22^*^	1.0 ± 0.14^*^	6.3 ± 0.19^*^	4.6 ± 0.16^*^	3.2 ± 0.19^*^	2.0 ± 0.17^*^
*ctaB*	9.4 ± 0.27	6.1 ± 0.12^*^	2.7 ± 0.16^*^	1.0 ± 0.15^*^	5.4 ± 0.13^*^	4.7 ± 0.17^*^	4.2 ± 0.31	2.9 ± 0.17^*^
*fbaA*	9.6 ± 0.12	8.1 ± 0.17	7.5 ± 0.16	6.0 ± 0.14	6.7 ± 0.25	6.1 ± 0.19	5.2 ± 0.13	4.0 ± 0.16
*clpX*	9.4 ± 0.17	7.5 ± 0.25	6.5 ± 0.17	4.8 ± 0.17	6.4 ± 0.17	5.1 ± 0.20	3.3 ± 0.32^*^	2.1 ± 0.27^*^
*mnhG1*	9.4 ± 0.25	7.5 ± 0.24	6.6 ± 0.15	5.1 ± 0.19	7.3 ± 0.13	5.6 ± 0.13	3.9 ± 0.15^*^	3.1 ± 0.19^*^
*parE*	9.6 ± 0.17	7.8 ± 0.26	6.8 ± 0.11	5.7 ± 0.16	7.1 ± 0.16	5.1 ± 0.18	3.9 ± 0.29^*^	3.5 ± 0.20^*^
*HOU_0223*	9.5 ± 0.16	7.5 ± 0.21	6.1 ± 0.21	4.2 ± 0.18	6.7 ± 0.20	6.3 ± 0.13	5.6 ± 0.16	3.9 ± 0.19
*HOU_0587*	9.6 ± 0.14	6.3 ± 0.15^*^	2.5 ± 0.64^*^	1.0 ± 0.17^*^	6.5 ± 0.13	5.3 ± 0.20	4.5 ± 0.26	4.2 ± 0.21
*HOU_2091*	9.7 ± 0.12	7.6 ± 0.38	7.3 ± 0.17	6.6 ± 0.16	7.4 ± 0.14	5.7 ± 0.12	4.1 ± 0.12^*^	3.4 ± 0.14^*^
*HOU_2315*	9.4 ± 0.13	7.5 ± 0.17	6.9 ± 0.20	5.8 ± 0.18	7.3 ± 0.14	5.6 ± 0.12	4.7 ± 0.13	3.9 ± 0.21
*HOU_2346*	9.5 ± 0.16	7.8 ± 0.23	6.9 ± 0.28	5.8 ± 0.16	6.6 ± 0.27	5.5 ± 0.17	4.8 ± 0.18	4.2 ± 0.21

Stationary-phase cultures of insertion mutants were evaluated for susceptibility to heat and acidity compared with the parent strain. For acid stress, cultures were harvested, washed, and then resuspended in TSB medium at pH 3.0. For heat stress, cultures were directly incubated at 57°C for various times. The number of CFU was determined by plating serial dilutions of bacteria on TSA plates.

* indicates significant differences in comparison to the parent strain (Student's t-test, P-value < 0.05).

### Decreased persister numbers in newly constructed *ureG, sdhA*, and *sdhB* knockout mutants in antibiotic exposure assays

**Figure 2:**
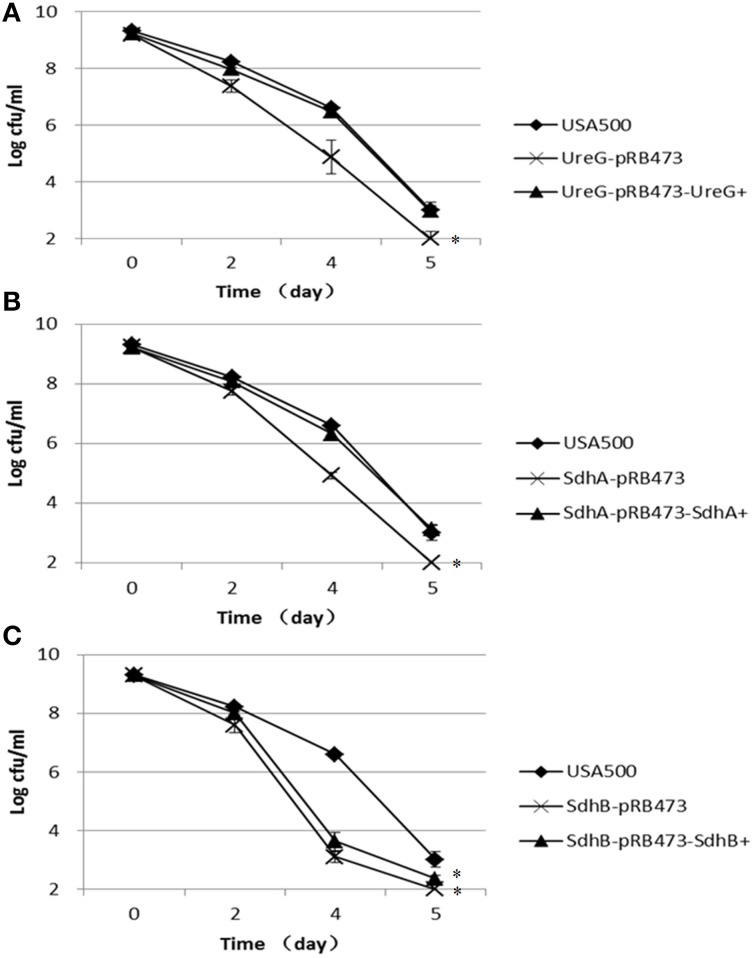
**Decreased persister levels of Δ*ureG*, Δ*sdhA*, Δ*sdhB* mutants in levofloxacin exposure assay**. Stationary-phase cultures of **(A)** Δ*ureG*, **(B)** Δ*sdhA*, and **(C)** Δ*sdhB* knockout mutants, along with their complemented strains and the wild-type strain USA500, were exposed to levofloxacin (12.5 μg/ml). Aliquots of cultures were taken at different time points, and the dilutions were plated for CFU determination on TSA plates. The error bars indicate standard deviations. ^*^indicates significant differences in comparison to the parent strain (*p*-value < 0.05).

To verify the role of the identified genes from the transposon mutant screening in persister formation or survival in *S. aureus* and to rule out potential polar effects of the transposon on the persister defect phenotype, we constructed fresh knockout mutants of the 3 candidate genes *ureG, sdhA*, and *sdhB* in *S. aureus* USA500 and their respective complements. When exposed to 12.5 μg/ml levofloxacin, the Δ*ureG*, Δ*sdhA*, and Δ*sdhB* strains showed decreased persister levels compared with the parent strain (Figure 2). The most significant decrease occurred after 4 days of exposure to levofloxacin in all mutants. The persister levels of *ureG, sdhA*, and *sdhB* mutants were decreased by 1.7-log, 1.7-log and 3.5-log, respectively, relative to the parent stain after 4 days of exposure. Complementation of the Δ*ureG* and Δ*sdhA* mutants with their respective wild-type genes rendered the persister levels close to that of the parent strain (Figure 2). However, complementation of Δ*sdhB* mutant only partially restored the the phenotype. These data indicate that *ureG* and*sdhA*, and to a lesser extent *sdhB*, are associated with persister formation under levofloxacin treatment in *S. aureus* USA500.

### The newly constructed *ureG, sdhA*, and *sdhB* knockout mutants are more susceptible to a variety of stresses

**Figure 3:**
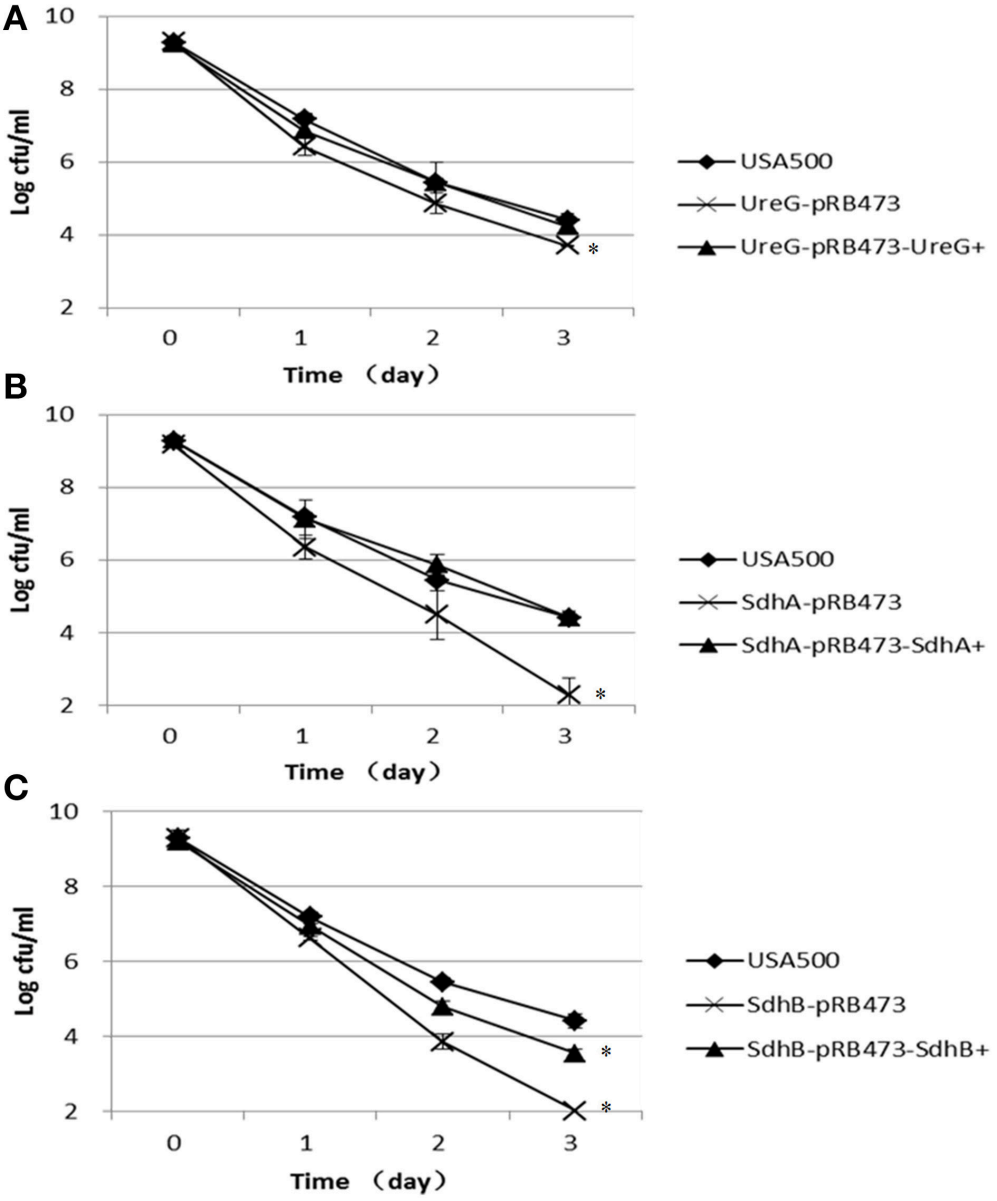
**Decreased persistence levels of Δ*ureG*, Δ*sdhA*, Δ*sdhB* mutants under acid stress (pH 3.0)**. Stationary-phase cultures of **(A)** Δ*ureG*, **(B)** Δ*sdhA*, and **(C)** Δ*sdhB* knockout mutants and their complemented strains were harvested, washed, and then resuspended in TSB medium at pH 3.0. The vertical axis represents CFU values in log scale, and the horizontal axis represents the time of the acid (pH 3.0) treatment in days. The error bars indicate standard deviations. ^*^indicates significant differences in comparison to the parent strain (*p*-value < 0.05).

**Figure 4:**
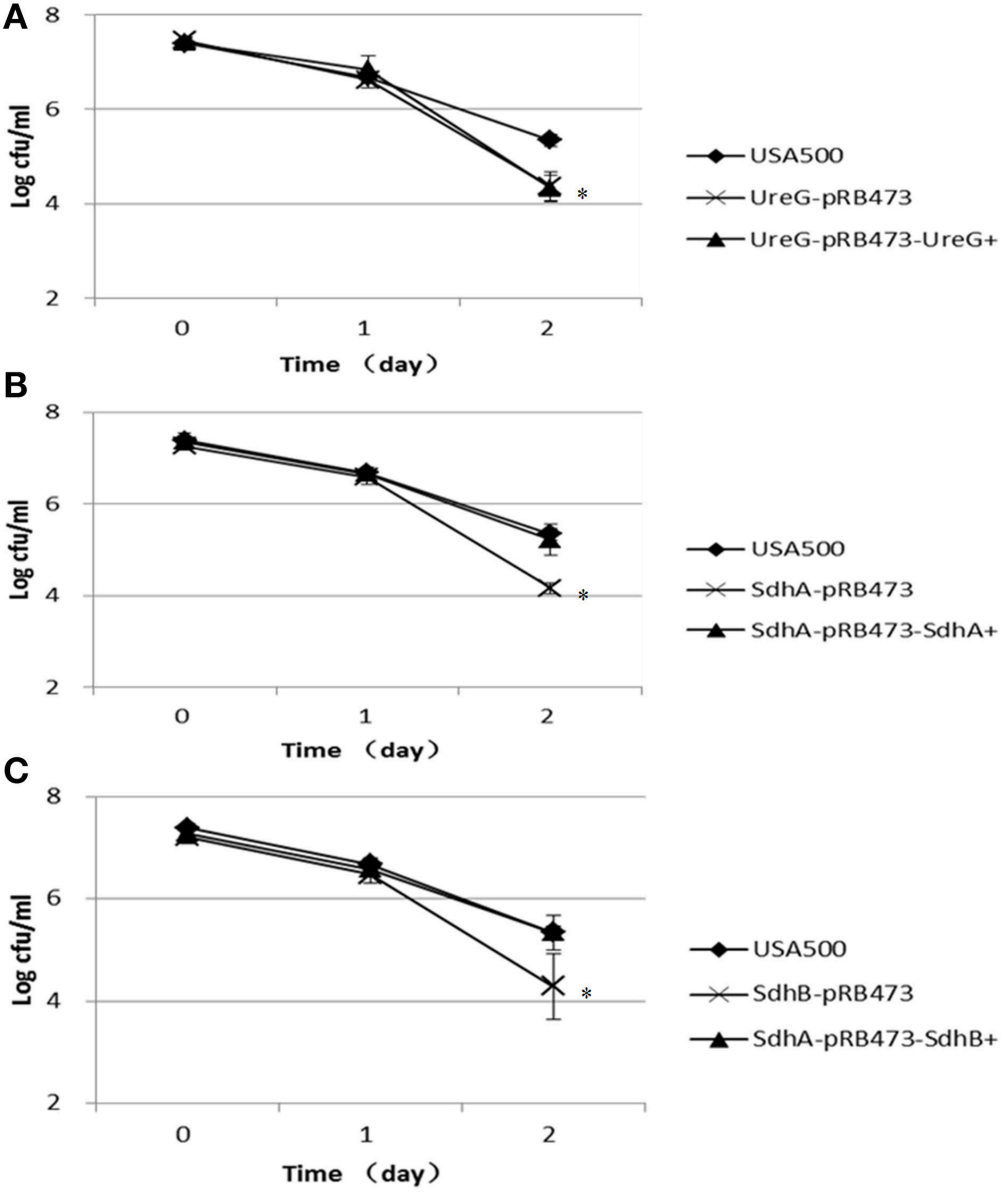
**Decreased persister levels in Δ*ureG*, Δ*sdhA*, Δ*sdhB* mutants under organic acid stress (acetic acid)**. Overnight cultures were diluted 1:100 in TSB medium containing 80 mM acetic acid. The parent strain USA500 was included as a control. The survival of the **(A)** Δ*ureG*, **(B)** ΔsdhA, and **(C)** Δ*sdhB* knockout mutants and their complemented strains was estimated at various time points of treatment. The error bars indicate standard deviations. ^*^indicates significant differences in comparison to the parent strain (*p*-value < 0.05).

Furthermore, we challenged these knockout strains with other stresses, including acid stress (inorganic acid, pH 3.0, and acetic acid, 80 mM, pH 4.4), oxidative stress (peroxide, 50 mM), and heat stress (57°C). Since *S. aureus* has versatile responses to HCL and organic acid stress, we chose both of the inorganic acid and organic acid for stresses assays (Rode et al., 2010). For the inorganic acid stress, the persister levels of Δ*sdhA* and Δ*sdhB* mutants were 2.1-log and 2.4-log lower than the parent strain, respectively, and Δ*ureG* showed a 0.7-log decrease compared with the parent strain after 3 days of treatment. Complementation of these mutants restored the complemented strains to the persister levels of the parent strains except Δ*sdhB* (Figure 3). The significant difference between Δ*sdhB* complementation strain and the parent strain indicated a partial complementation. The organic acetic acid stress results were different from those for the above inorganic acid, with the three knockout mutants showing only 1-log decreases in persister levels compared with the parent strain USA500 after 2 days of treatment. The persister levels of the complementation strains were close to those of the parent strain except in the *ureG* complementation mutant (Figure 4).

**Figure 5:**
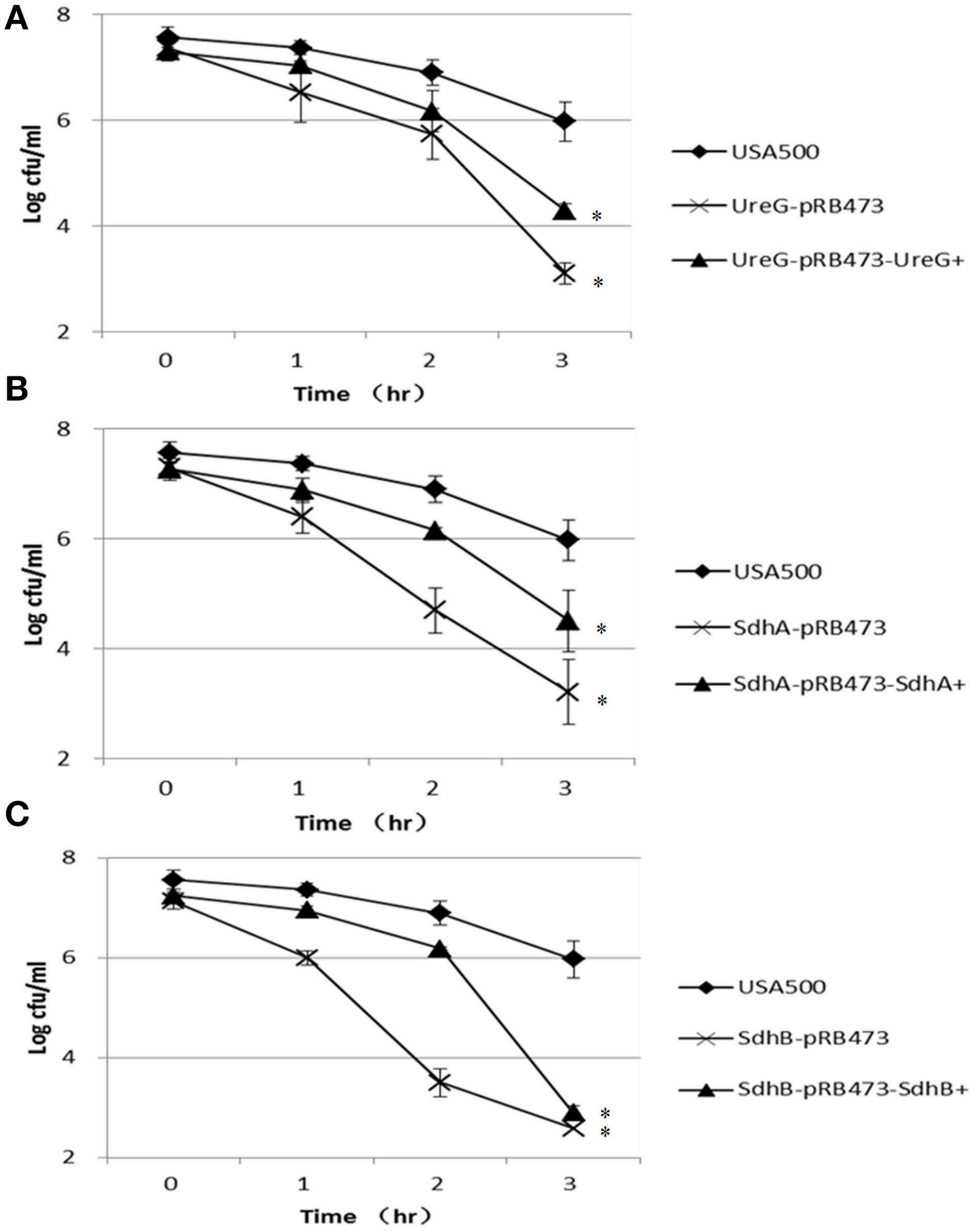
**Decreased persister levels of Δ*ureG*, Δ*sdhA*, and Δ*sdhB* mutants under oxidative stress (hydrogen peroxide)**. Stationary-phase cultures were diluted 1:100 in TSB medium. Hydrogen peroxide was added to a final concentration of 50 mM. The susceptibilities of the **(A)** Δ*ureG*, **(B)** Δ*sdhA*, and **(C)** Δ*sdhB* knockout mutants and their complemented strains were determined. The error bars indicate standard deviations. ^*^indicates significant differences in comparison to the parent strain (*p*-value < 0.05).

In the peroxide exposure (50 mM H_2_O_2_) assay, the persister levels of Δ*ureG*, Δ*sdhA*, and Δ*sdhB* mutants were 3.0-log, 2.8-log, and 3.4-log lower than that of the parent strain, respectively, after 3 h of treatment. Except for the *sdhB* mutant, the persistence of these mutants could be partially complemented (Figure 5).

**Figure 6:**
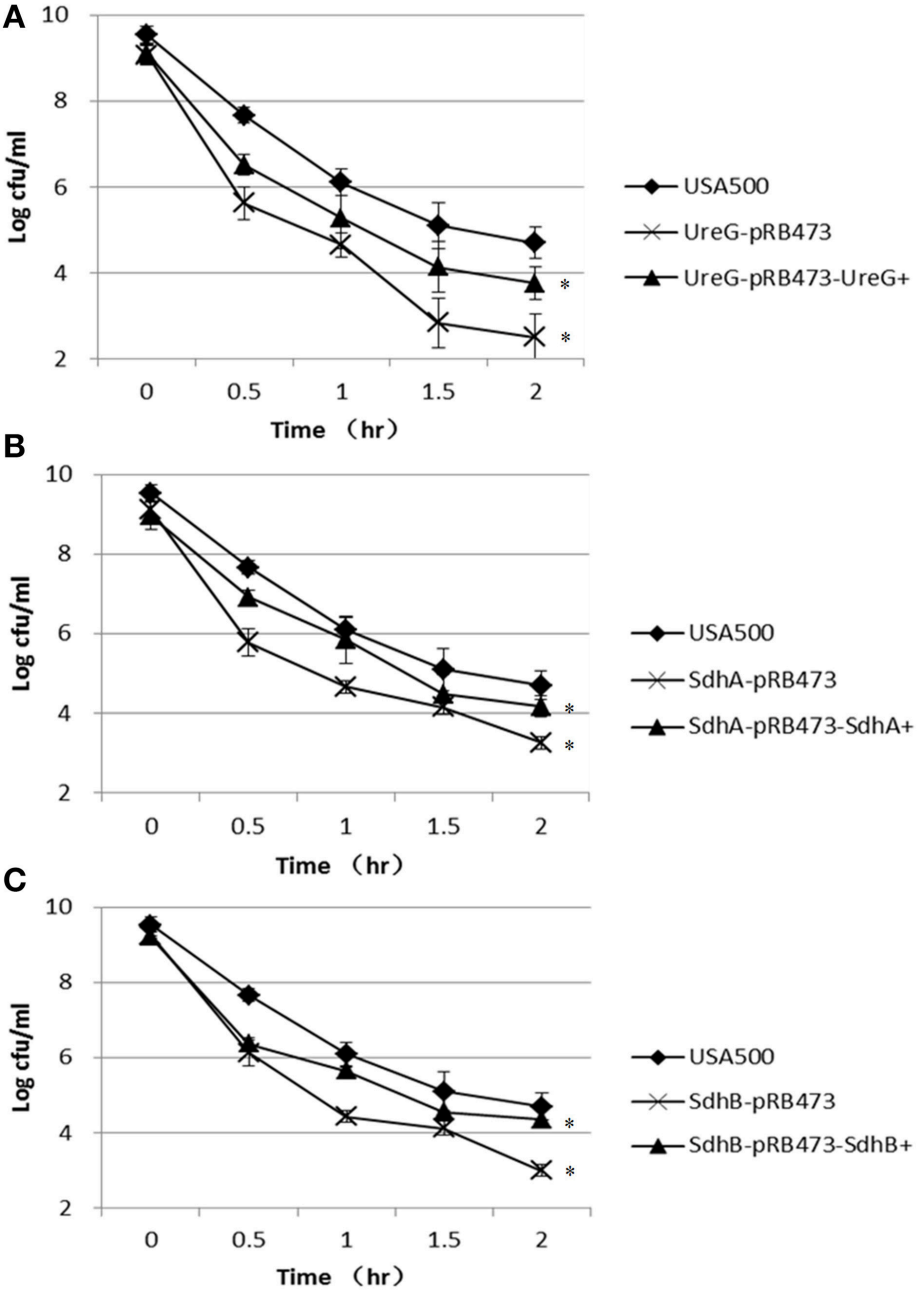
**Decreased persister levels of Δ*ureG*, Δ*sdhA*, and Δ*sdhB* mutants under heat stress (57°C)**. Stationary-phase cultures of the **(A)** ureG, **(B)** sdhA, and **(C)** sdhB mutants, complemented strains and the parent strain were incubated at 57°C for various times, and the number of CFU was determined by plating serial dilutions of bacteria on TSA plates. The error bars indicate standard deviations. ^*^indicates significant differences in comparison to the parent strain (*p*-value < 0.05).

In the heat stress (57°C) assay, the Δ*ureG*, Δ*sdhA*, and Δ*sdhB* mutants were also more susceptible to the heat stress, showing decreased persistence by 2.2-log, 1.4-log, and 1.7-log, respectively, compared with the parent strain after 2 h of treatment. However, complementation only partially restored thepersister levels of Δ*ureG*, Δ*sdhA*, and Δ*sdhB* mutants (Figure 6).

## Discussion

In this study, we identified 13 genes that could be associated with persister formation or survival in *S. aureus*. The inactivation by insertion mutation led to increased susceptibility to levofloxacin and other stresses. Because of the difficulty and large amount of work involved in validating all 13 genes, in this study we focused on validating the *ureG, sdhA*, or *sdhB* genes by constructing new knockout mutants, which were identified in multiple insertion mutants and in more than one insertion site, suggesting a strong possibility that these genes are involved in persister formation in *S. aureus* USA500. Another reason to construct new knockout mutants is to rule out the polar effect of transposon insertion on downstream genes. The finding that the freshly constructed *ureG, sdhA*, and *sdhB* knockout mutants also had the same decreased persister numbers in antibiotic exposure assay as the transposon mutants demonstrated that these genes are indeed involved in persister formation. Furthermore, complementation of these genes could at least partially restore the persistence phenotypes. *UreG, sdhA*, and *sdhB* are single genes located in a large operon. Once the operon is disrupted, gene expression may be different in genome vs. plasmid-based complementation. Additionally, the influence of other genes in the plasmid and possible compensation by other genes in the genome may also be the reasons for partially restoration. Further studies are needed to validate other insertion mutants by gene knockout in future studies.

To the best of our knowledge, this is the first comprehensive study using a transposon mutant library screen to systematically identify persister genes in *S. aureus*. Our study provides important insights into the mechanisms of persister formation in *S. aureus* and identifies new drug targets for persister-targeted antibiotic development for the improved treatment of chronic and persistent infections.

Several genes that are involved in energy production in *S. aureus* were identified in our study. The gene *fbaA* encodes fructose-bisphosphate aldolase, which is involved in bacterial glycolysis. The *sdhA, sdhB*, and *sdhC* genes compose the *sdhCAB* operon and encode succinate dehydrogenase in *S. aureus*, which is essential in the non-oxidative branch of the tricarboxylic acid (TCA) cycle. Our finding is consistent with previous studies in *E. coli* that showed that energy production genes, such as *sucB, ubiF*, and *acnB*, are involved in persister formation (Ma et al., 2010; Luidalepp et al., 2011). In addition, genes that are involved in the TCA cycle have been demonstrated to be upregulated in *S. aureus* biofilms; these include genes of the *sdh* operon, 2-oxoglutarate dehydrogenase (*odhA*), and succinyl-coenzyme A (CoA) synthetase (*surC*) (Resch et al., 2005, 2006), which may link their role in biofilms to persister formation.

Multiple clones and transposon insertion sites were identified in the *sdhA, sdhB, HOU_0587*, and *ctaB* genes among the persister-defective mutants in our study, which suggests that the electron transport chain may be involved in persister formation. The multiple clones that were identified in multiple insertion sites of these genes strengthen the credibility of these results and provide strong support for their role in persistence. The *sdh* operon consists of three genes, which encode a flavoprotein that contains an FAD binding site (SdhA), an iron-sulfur protein with a 4Fe-4S-type binding region (SdhB) and membrane-bound cytochrome *b*_588_ that contains two binding sites for heme (SdhC). The succinate dehydrogenase catalyzes the oxidation of succinate to fumarate and produces FADH_2_ for oxidative phosphorylation (Hederstedt and Rutberg, 1980, 1981). A previous study confirmed that ROS or free radicals produced by the electron transport chain lead to bacterial death under oxidative stress (Mols and Abee, 2011). In addition, the electron transport chain is associated with drug tolerance and virulence in *S. aureus* small colony variants (SCVs). SCVs have characteristic deficiencies in the electron transport chain that mainly consist of heme- and menaquinone-synthesis deficiency. Compared with wild-type levels, the persister numbers in *hemB* and *menD* mutants were significantly decreased at high concentrations of antibiotics (Lechner et al., 2012a). Mutations in other genes involved in the heme and menaquinone pathways also display SCV phenotypes, including *ctaA, menB, hemA, hemH, mutS, fusA*, and *thyA* (Clements et al., 1999; Schaaff et al., 2003; Besier et al., 2007). Meanwhile, the *sdhCAB* knockout mutant showed an SCV-like phenotype (Gaupp et al., 2010). These results indicate that the electron transport chain may play an important role in persister formation in *S. aureus*. Our findings that *sdhA, sdhB, HOU_0587*, and *ctaB* are involved in persister formation are consistent with the observation of the SCV persister phenotypes. Future studies are needed to determine the detailed mechanisms involved.

The *ureG* mutant also displayed a significant decrease in persister numbers after exposure to a variety of stresses. The mechanism of persister deficiency in the *ureG* mutant is unknown. The *ureG* gene encodes a subunit of urease. Ureases consist of several subunits, including structural proteins (UreA, UreB, and UreC) and accessory proteins (UreD, UreE, UreF, and UreG), and are widely present in bacteria, fungi, plants, and invertebrates. Studies have demonstrated that urease is important for bacterial survival at low pH in various organisms including *Helicobacter pylori, Klebsiella pneumonia*, and *Yersinia enterocolitica* (Marshall et al., 1990; Young et al., 1996; Maroncle et al., 2006). Urease is also associated with virulence in *S. aureus* and *Brucella abortus* (Highlander et al., 2007; Sangari et al., 2007). These studies indicate that the *ureG* mutant may influence *S. aureus* virulence by affecting the activity of urease. The exact role of *ureG* in persister formation needs to be determined in further studies.

ClpX is a member of the Clp proteolytic complex, and its deficiency reduces biofilm formation (Frees et al., 2004). A previous study demonstrated that persisters exist in biofilm-associated *S. aureus* (Singh et al., 2009). Our findings indicate that *clpX* plays an important role in persister and biofilm formation in *S. aureus*, although the mechanisms need to be further studied. The *parE* gene encodes subunit B of DNA topoisomerase IV; this enzyme is involved in DNA repair, which is known to be involved in persister formation or survival in *E. coli* (Debbia et al., 2001).

In addition to genes from pathways that are known to be involved in persister formation and survival, genes involved in cell transport were identified, including *mnhG1* and *HOU_0223*. However, knockouts of these genes did not show obvious reductions in persister numbers (data not shown). The insertion transposon may partially disrupt the operon, and the partially functional genes may have different functions and therefore display different phenotypes than those associated with the wild-type genes. If the genes are deleted, the transport or sensor functions may be compensated by other genes or pathways. In this way, the reconstructed deletion mutants may lose the obvious phenotype or even acquire an opposite phenotype. For example, the *phoU* deletion mutant can be compensated by mutation in *phoR*, such that the *phoU* mutant does not show obvious defect in persister levels (Shi et al., unpublished), whereas the *phoU* transposon mutant has an obvious persister defect (Li and Zhang, 2007).

One limitation of the study is the gene coverage of the transposon mutant library. The multiple independent mutant hits for the top hits (Table 1) while reassuring in confirming the role of these genes in persister phenotype, may suggest a potential bias in the mutant library. In fact, to avoid the transposon integration bias of the previously used transposon, we used the mariner-based plasmid for transposon mutagenesis. In our previous virulence studies with the same transposon mutant library, no apparent bias for mariner transposon integration was found (Li et al., 2009). However, multiple insertions in *HOU_2091* and *HOU_2315* mutants indicate the possibility of a bias of the mutant library in this study (Table 1). Thus, certain genes that are important in persister formation and survival may have been missed in this study. Future studies with more clones or with different mutant libraries may be needed to more comprehensively identify *S. aureus* persister genes.

In summary, we identified 13 novel genes which may associated with persister survival and formation in *S. aureus.* These genes are involved in several pathways including energy production, electron transport chain, DNA repair, biofilm formation, and cell transport. These findings provide novel insights into the mechanisms of persister formation and survival in *S. aureus*, and offer new targets for developing new antibiotics that kill persisters for improved treatment of chronic and persistent infections.

## Author contributions

YZ, ML, and WZ designed the work and revised the manuscript; WW, JC, GC, XD, and PC completed all the experiments; JW, JZ, and NW performed the statistically analysis and made the figures; WW and JC, YZ wrote the manuscript.

### Conflict of interest statement

The authors declare that the research was conducted in the absence of any commercial or financial relationships that could be construed as a potential conflict of interest.
