# Supplementary material for: Transposon Mutagenesis Identifies Novel Genes Associated with Staphylococcus aureus Persister Formation
Source: Front Microbiol. 2015 Dec 23;6:1437. doi: 10.3389/fmicb.2015.01437 (PMC4689057; doi:10.3389/fmicb.2015.01437)
Supplement: Supplementary file 1 [file Table1.PDF]

TABLE S1. Drug susceptibility test results of the parent strain *S. aureus* USA500.

Drugs	Drug susceptibility	Inhibition zone（mm）
Gentamicin	S[a]	22
Penicillin	R[b]	10
Oxacillin	R	6
Cefazolin	R	9
Cefuroxime	R	6
Cefoxitin	R	16
Vancomycin	S	27
Linezolid	S	28
Levofloxacin	S	27
Erythromycin	S	27
Clindamycin	S	26
Fosfomycin	S	30
Rifampicin	S	30
Teicoplanin	S	16

a The USA500 strain was susceptible to the drug.

b The USA500 strain was resistant to the drug.
