# Supplementary material for: Transposon Mutagenesis Identifies Novel Genes Associated with Staphylococcus aureus Persister Formation
Source: Front Microbiol. 2015 Dec 23;6:1437. doi: 10.3389/fmicb.2015.01437 (PMC4689057; doi:10.3389/fmicb.2015.01437)
Supplement: Supplementary file 2 [file Table2.pdf]

TABLE S2. The PCR primers used in this study.

Primer name	Sequence (5'to3')
erm-5.1	GCTTCTAAGTCTTATTTCCATAAC
erm-5.3	TCTACATTACGCATTTGGAATAC
erm-3.1	TAGGTATACTACTGACAGCTTC
erm-3.3	TACTTATGAGCAAGTATTGTCTA
arb-1	GGCCACGCGTCGACTAGTCANNNNNNNNNNGATAT
arb-3	GGCCACGCGTCGACTAGTCA
ureG-att1	ggggacaagtttgtacaaaaaagcaggctAAATTCAGAAAAACAAAAGCACGTCG
ureG-rev1	TTGTTCATCCAAATTATAACCTCCTATGATATGAAAATTCTAACA
ureG-rev2	CATATCATAGGAGGTTATAATTTGGATGAACAACAATGGACTGGG
ureG-att2	ggggaccactttgtacaagaaagctgggtGGTTAATTGCGTTTAATAACATTGAACGA
sdhA-att1	ggggacaagtttgtacaaaaaagcaggctCAAACACGTCAAAAGACAGGCTTG
sdhA-rev1	AAAAATTTCACTCCCCTAAATTTTCAA
sdhA-rev2	TAGGGGAGTGAAATTTTTTGACTGAACAATCAGTGAAAAACACTCC
sdhA-att2	ggggaccactttgtacaagaaagctgggtCCATTAATTTACGAGCGATTTCAACT
sdhB-att1	ggggacaagtttgtacaaaaaagcaggctTATTATGGCAACGGGTGGCCC
sdhB-rev1	TATTTTTTACCCCCTTTAGACTTACTTGT
sdhB-rev2	CTAAAGGGGGTAAAAAATAAAATTAATCCCTTTTGAGTTAAAATGTTATG
sdhB-att2	ggggaccactttgtacaagaaagctgggtCAATGCTTTTGCAGCAGCTTCTG
ureG-Pro-F	GCAGAATTCCCTTTCCTTTGAATCATTACTCA
ureG-Pro-linker	GATTTGCCACAATTAAAACCCCCAATTTCATAT
ureG-gene-linker	GGGTTTTAATTGTGGCAAATCCGATTAAAATT
ureG-gene-R	ACGGGATCCTTATGATAATCCTTTGAGTAAAGTG
sdhAB-Pro-F	ATTAGAGCTCTACTGGGCGTGAGAATCATTCT
sdhAB-Pro-linker	TAAATGCGATGTAAAGGAGTCCCCCCTGTGAA
sdhAB-gene-linker	GGGGACTCCTTTACATCGCATTTAACAAATATAGATTG

sdhAB-gene-R	TACAGTCGACTAATCTGCTTATTAAAAAAATCCCTT
