# Supplementary material for: Transposon Mutagenesis Identifies Novel Genes Associated with Staphylococcus aureus Persister Formation
Source: Front Microbiol. 2015 Dec 23;6:1437. doi: 10.3389/fmicb.2015.01437 (PMC4689057; doi:10.3389/fmicb.2015.01437)
Supplement: Supplementary file 3 [file Image1.pdf]

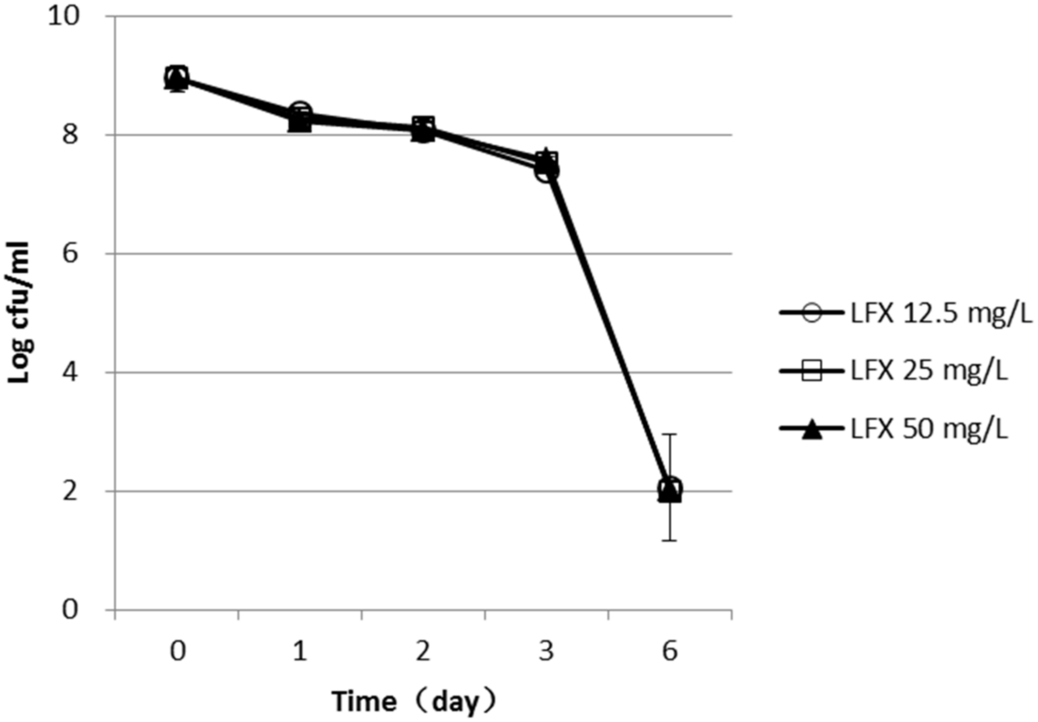

Figure S1. Persister levels of the parent *S. aureus* strain USA500 at different concentrations of levofloxacin in the persister assay. The stationary-phase culture of the parent strain USA500 was exposed to different concentrations of levofloxacin (LFX), at 12.5, 25 and 50 μg/ml and incubated at different times. The survival of the persisters was monitored by CFU count after washing and plating on TSA plates. The vertical axis represents CFU values in log scale, and the horizontal axis represents the time of exposure. The error bars indicate standard deviations.
