# Supplementary material for: Transposon Mutagenesis Identifies Novel Genes Associated with Staphylococcus aureus Persister Formation
Source: Front Microbiol. 2015 Dec 23;6:1437. doi: 10.3389/fmicb.2015.01437 (PMC4689057; doi:10.3389/fmicb.2015.01437)
Supplement: Supplementary file 4 [file Image2.pdf]

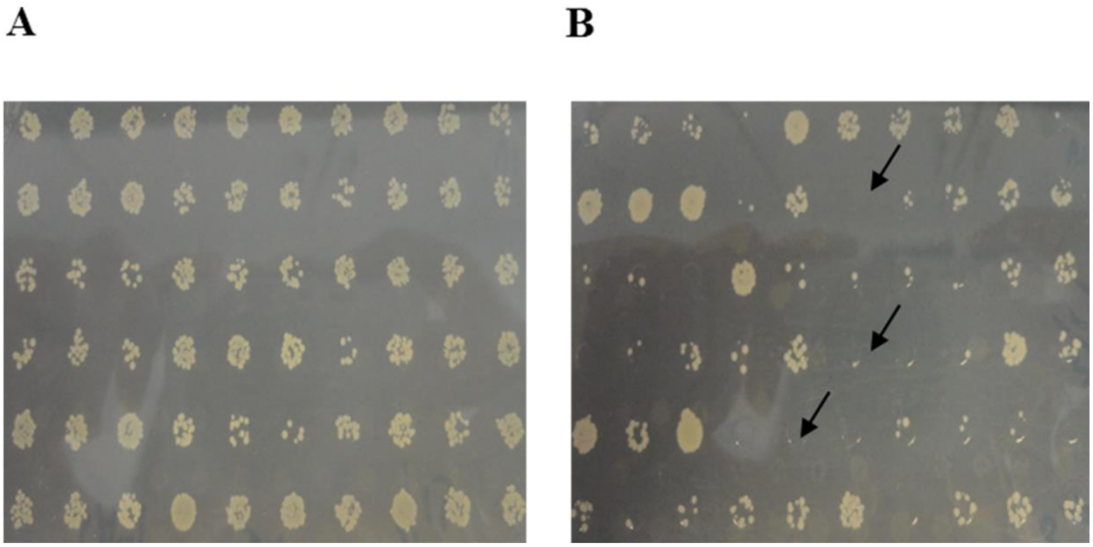

Figure S2. The transposon library of *S. aureus* USA500 was screened for persister deficiency mutants using persister assays with levofloxacin. Stationary-phase cultures of the transposon mutant library were exposed to 12.5 μg/ml levofloxacin for 6 days. The library was replica-transferred onto TSA plates to compare the levels of surviving bacteria after 3 days (A) and 6 days (B) of drug exposure at 37 ℃. The arrows indicate selected representative mutants with obvious persister deficiency compared with the parent strain USA500.
